# Impairment of organ-specific T cell negative selection by diabetes susceptibility genes: genomic analysis by mRNA profiling

**DOI:** 10.1186/gb-2007-8-1-r12

**Published:** 2007-01-21

**Authors:** Adrian Liston, Kristine Hardy, Yvonne Pittelkow, Susan R Wilson, Lydia E Makaroff, Aude M Fahrer, Christopher C Goodnow

**Affiliations:** 1 John Curtin School of Medical Research, The Australian National University, Canberra, ACT 2601, Australia; 2 Mathematical Sciences Institute, The Australian National University, Canberra, ACT 2601, Australia; 3 Biochemistry and Molecular Biology, The Australian National University, Canberra, ACT 2601, Australia; 4 The Australian Phenomics Facility, The Australian National University, Canberra, ACT 2601, Australia; 5 Department of Immunology, University of Washington, Seattle, WA 98195, USA

## Abstract

**Background:**

T cells in the thymus undergo opposing positive and negative selection processes so that the only T cells entering circulation are those bearing a T cell receptor (TCR) with a low affinity for self. The mechanism differentiating negative from positive selection is poorly understood, despite the fact that inherited defects in negative selection underlie organ-specific autoimmune disease in *AIRE*-deficient people and the non-obese diabetic (NOD) mouse strain

**Results:**

Here we use homogeneous populations of T cells undergoing either positive or negative selection *in vivo *together with genome-wide transcription profiling on microarrays to identify the gene expression differences underlying negative selection to an *Aire*-dependent organ-specific antigen, including the upregulation of a genomic cluster in the cytogenetic band 2F. Analysis of defective negative selection in the autoimmune-prone NOD strain demonstrates a global impairment in the induction of the negative selection response gene set, but little difference in positive selection response genes. Combining expression differences with genetic linkage data, we identify differentially expressed candidate genes, including *Bim*, *Bnip3*, *Smox*, *Pdrg1*, *Id1*, *Pdcd1*, *Ly6c*, *Pdia3*, *Trim30 *and *Trim12*.

**Conclusion:**

The data provide a molecular map of the negative selection response *in vivo *and, by analysis of deviations from this pathway in the autoimmune susceptible NOD strain, suggest that susceptibility arises from small expression differences in genes acting at multiple points in the pathway between the TCR and cell death.

## Abstract 2

A transcription profiling study, together with genetic linkage data, provides a molecular map of the T-cell negative selection response *in vivo*.

## Background

Immunological self-tolerance depends upon negative selection in the thymus, whereby T cells bearing T cell receptors (TCRs) with high avidity for self peptide-major histocompatibility complex (MHC) complexes are purged from the developing repertoire before they become functionally active in the periphery [1]. Negative selection occurs by TCR-induced apoptosis during the transition of immature CD4^+^8^+ ^double positive (DP) cells into mature CD4^+ ^or CD8^+ ^single positive (SP) cells. Nevertheless, a measure of TCR signaling is required for thymocytes to mature from DP into SP cells, an opposite process requiring a weak avidity for self peptide-MHC in order to initiate the changes of cell survival and maturation referred to as positive selection. It is thought that these two opposite processes, of cell survival or death, initiated by binding of the same receptor to its ligand, are controlled by quantitative differences in TCR affinity for self peptide-MHC that are translated into qualitatively opposite cellular responses. However, the molecular basis by which the two processes are differentially controlled and how the cellular responses initiated are achieved are unclear [2].

Recent studies have found that inherited defects in negative selection underlie autoimmune disease. In the human disorder autoimmune polyendocrinopathy syndrome type 1, defects in the *AIRE *gene reduce transcription of organ-specific genes in the thymus so that organ-reactive T cells are not negatively selected [3-5]. The non-obese diabetic (NOD) mouse strain is an intensely studied model for human autoimmune diabetes, as well as being susceptible to other autoimmune disorders [6], and displays a striking cellular deficiency in MHC class II- [7,8] and class I-restricted negative selection [9], compared to non-autoimmune-prone strains. Elucidating the molecular basis for defective negative selection in NOD mice may shed light on the process of differentiating negative from positive selection and the pathogenesis of human autoimmunity.

The NOD thymic deletion defect is T cell autonomous [7,8] and represents a quantitative (approximately ten-fold) decrease in negative selection efficiency to membrane-bound or soluble proteins, regardless of low or high thymic expression controlled by organ-specific or systemic promoters [10]. Genetic linkage studies between the C57BL10.*H2*^k ^(B10^k^) and NOD strains identified four NOD-derived recessive loci that contribute to defective negative selection *in vivo*, by tracing CD4 T cell deletion triggered in the thymus of transgenic mice by expression of an *Aire*-dependent antigen (insHEL) that mirrors the expression of the insulin gene itself [10]. As would be expected, the four defective deletion loci correspond to four NOD loci known to contribute to diabetes susceptibility, linked to the markers *D7mit101*, *D15mit229*, *D2mit490/Idd13 *and *D1mit181/Idd5*. Parallel analyses *in vitro*, in a thymic organ culture system with exogenously added antigen, found NOD loci that acted dominantly to interfere with apoptosis linked to two recessive diabetes susceptibility loci, *Idd5 *and *Idd3 *[11].

Gene expression profiling on microarrays provides an opportunity to visualize the molecular differentiation of negative from positive selection and defects in negative selection at a global level. Several key questions can, in principle, be addressed. First, does negative selection involve induction or repression of a unique set of genes, or simply quantitatively exaggerated changes in the same set of genes as positive selection? Does the negative selection defect in NOD mice interfere with all or only a small number of negative selection genes, or does it cause a new profile of counter-regulatory genes to be triggered, and does it equally affect positive selection gene responses? Several studies have begun to explore this approach, although they have been limited by complications, such as premature negative selection at the double negative (DN) to DP transition, pooling of developmental subsets, TCR heterogeneity, and the peripheral cytokine storm that is produced after cognate antigen injection [12-15].

Here we extend a preliminary published analysis [10] using an approach that provides a unique opportunity to visualize the global expression changes under physiological *in vivo *conditions of negative and positive selection. The model employs TCR transgenic mice to trace selection of T cells with a homogeneous TCR recognizing a well-characterized high affinity peptide-MHC agonist, hen egg lysozyme (HEL) 46-61/I-A^k^. This TCR is expressed at low levels on DP cells, and promotes positive selection into TCR^hi ^CD4^+ ^SP cells following the physiological pathway and induction of markers such as CD69 and CCR7 [10]. When the TCR transgenic is crossed to insHEL transgenic mice on the non-autoimmune B10^k ^strain background, the insulin-promoter activates *Aire*-dependent transcription to produce low levels of HEL antigen in thymic medullary epithelial cells, triggering negative selection at the physiological stage during the transition from cortical DP cells to medullary SP cells. Unlike thymocyte apoptosis initiated through the intravenous injection of antigen or anti-TCR antibodies, deletion of HEL-reactive thymocytes by endogenous presentation of HEL activates a physiological apoptotic process that is T cell intrinsic, with efficient deletion of HEL-reactive thymocytes at various dilutions of precursor frequency and no apoptosis of neighboring non-HEL-specific thymocytes (data not shown). This system of negative selection also has the advantage of faithfully replicating the expression pattern of a key autoantigen in human disease, insulin. By comparing global gene expression in homogeneous subsets of sorted cells from these mice, either pre-selection or undergoing positive or negative selection, we present here a detailed analysis of the gene expression differences distinguishing negative from positive selection in a non-autoimmune prone strain, and analyze the underlying defect in negative selection in the autoimmune NOD strain.

## Results and discussion

### Global gene expression changes differentiating positive and negative thymocyte selection in the B10^k ^strain

To characterize the gene expression changes occurring during positive and negative selection in a non-autoimmune strain, gene expression profiles were measured in three relatively homogeneous thymocyte populations from the B10^k ^strain [10]. Pre-selection thymocytes ('PreS') that had not yet received positive or negative selection signals were identified as CD4^+^8^+ ^DP cells that are negative for CD69 and 1G12 TCR clonotype and sorted from TCR transgenic and TCR insHEL double transgenic mice. Early single positive cells that were beginning to undergo positive selection (S+) were sorted from TCR transgenic mice as CD4^+^CD8^low^CD69^+^1G12^+ ^cells. Early single positive cells with an identical cell surface phenotype but beginning to undergo negative selection (S-) were sorted from TCR insHEL double transgenic animals. Three independent pools of mRNA from sorted cells were analyzed by labeling and hybridization to Affymetrix 430A microarrays and normalized using MAS 5.0. MAS 5.0 was used as the normalization method in preference to more precise project-based model fitting systems, such as RMA, GCRMA and PLM, due to advantages in simple reduction of the false discovery rate. Use of the MAS 5.0 normalization method produces 'present' and 'absent' calls and, when used as a filtering device, this has been demonstrated to reduce the false discovery rate with little true cost to the true positive rate [16]. Furthermore, by taking the mismatch probe into consideration, MAS 5.0 reduces the level of false positives based on cross-hybridization [16]. It should be noted that all normalization methods involve a trade-off of accuracy or precision at various levels and, while MAS 5.0 performs strongly for background correction and medium and high intensity signals [17], it is less accurate than some alternatives for probesets in the low intensity range [18,19]. The complete dataset, with raw values and statistical analysis is given in Additional data file 1 and has been deposited in the NCBI Gene Expression Omnibus (GEO) [20] accessible through GEO Series accession number GSE3997.

**Figure 1:**
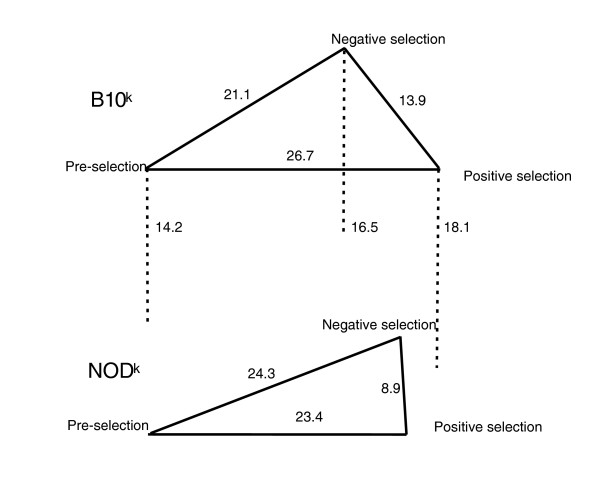
Global gene expression differences induced upon positive or negative selection on the B10^k ^and NOD^k ^genetic backgrounds. The global difference in gene expression between conditions was calculated as a Euclidean distance, taking into account the number of genes with differential expression, and the scale of differential expression, for each replicate chip. Euclidean distances are represented as the distance between conditions for both the B10^k ^and NOD^k ^genetic backgrounds, with the average positions of groups located at the apexes of the triangles. Dotted lines correspond to the Euclidean distance between B10^k ^and NOD^k ^genetic backgrounds, for the same population.

We first performed a global analysis of the differences in gene expression between negative and positive selection and pre-selection by measuring the Euclidian distance between conditions (Figure 1). Measuring Euclidian distance involves treating each condition as a single point in n-dimensional space, where each dimension is the expression of a single probeset. The distance between two conditions can then be calculated as the straight line ('ordinary') distance between the two points (conditions), so that, for example, a low value between two conditions indicates that they have similar values for gene expression, while a high value between two conditions indicates that they have different values for gene expression [21]. The advantage of Euclidian distance is that it is an approximation of the closeness of global gene expression profiles under different conditions, independent of classification of gene changes as significant or non-significant by including the number of genes that are unchanged, and the degree of change in differentially expressed genes (thus allowing subtle changes in large numbers of genes, which would not necessarily be detected if a statistical threshold was required, to impact global distance). Using Euclidean distance as the measure of global 'closeness', the largest difference was between pre-selection thymocytes and selecting thymocytes (both positive and negative selection). Negative selection was closer to the pre-selection condition than positive selection.

**Figure 2:**
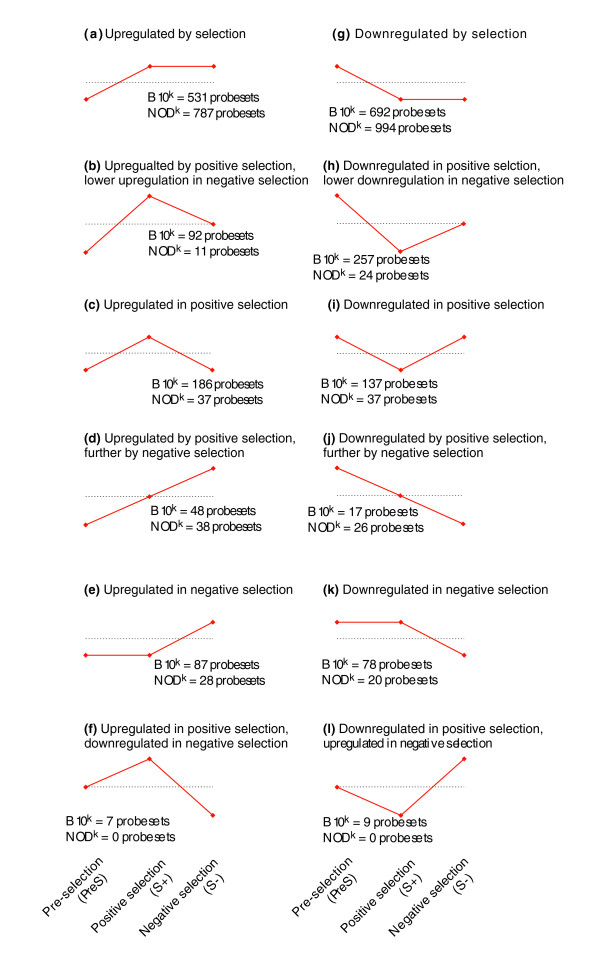
Patterns of gene expression changes induced by positive and negative selection in the B10^k ^and NOD^k ^strains. Analysis of Affymetrix GeneChip data segregated those probesets that were significantly changed upon positive and/or negative selection (*p *< 0.005) into patterns A to F based on logical sets of significant contrasts (*p *< 0.05), where patterns are defined by the relative expression during pre-selection (PreS, left), positive selection (S+, middle) and negative selection (S-, right). The number of probesets falling into each pattern in B10^k ^and NOD^k ^mice is listed on the expression pattern. Assignment of probesets to patterns was based only on direction of change, thus the graphical depictions do not represent the degree of change.

Individual gene expression changes were then analyzed in an independent method of assessment, by assigning probesets into categories based on statistical significance of gene expression pattern in the 3 replicates, allowing categorization into 12 possible patterns. Assignment to the 12 patterns was based only on the direction of significant gene expression change rather than degree of change (Figure 2). Global expression 'closeness' could then be estimated by comparing the number of probesets that fit each category.

This measure of global expression 'closeness' between conditions gave a result consistent with the Euclidean analysis, where the pattern categories with the largest number of probesets were those (patterns A and G in Figure 2) displaying an expression difference between pre-selection and selection (both positive and negative selection) but no difference between positive and negative selection. These are likely to represent genes that are developmentally regulated as part of the differentiation of immature DP cells into more mature early SP cells. Some may be induced or repressed by TCR signaling mechanisms that are unable to distinguish between TCR engagement by weak positively selecting agonists and the strong negatively selecting agonist. Pattern A comprised 531 probesets that were induced during maturation/selection, and pattern G comprised 692 probesets that were repressed. The induced genes included well established markers and mediators of maturity, *Tcr*, *Ccr7*, *S1P1 *and *IL7R*, and genes that are known to be targets of positive and negative selection TCR signals, such as the calcineurin-response genes *Ian1*, *Egr2 *and *CD52 *and ERK-response genes *Nab2 *and *Zfp36l1*. The repressed genes included *Rag1*, *preTαβ*, *CD8α *and *CD8β*, known to be involved in thymocyte maturation changes, and cell cycle genes *Cdc7*, *Cdk2*, *Cdk2ap1*, and *Cdk4*, which are consistent with the exit from cell cycle that accompanies maturation of early DP cells, which are cycling, into later DP and SP cells, which are non-cycling. The identification of these expected expression changes acts as an internal validation of the dataset.

As well as these previously identified markers of positive and negative selection, several important T cell regulatory genes were also found here to be upregulated in selection: those encoding nucleic acid binding proteins, such as *Bcl3 *[22], *Dicer1*[23], *Fosb *[24], *Hivep1 *[14], *Irf1 *[25], *Irf4 *[14], *Irf7 *[26], *JunB *[27], *Klf2 *[28], *Nfat5 *[29], *Rora *[30], *Stat1 *[13], and *Stat6 *[31]; signaling-associated genes *Ccnd2 *[14], *Evl *[32], *Hspa5 *[33], *Ly9 *[34], *Mcl1 *[35], *Ndfip1 *[33], *Psen2 *[36], *Rassf5 *[37], *Upf2 *[38] and *Zfpn1a *[39]; and those encoding receptors, such as *Crry *[40], *Gpr83 *[41], *2-K1 *[42], *Ms4a6b *[43], *Sema4a *[43], *Slamf1 *[43], *Tlr1 *[44], *Tnfsf11 *[45], and *Tslpr *[46].

Previously unassociated genes identified as part of this analysis are, therefore, excellent candidates for novel maturation markers, and include those encoding nucleic acid binding proteins, such as *1810007M14Rik*, *AA408868*, *Aptx*, *Arts1*, *D11Lgp2e*, *D1Ertd161e*, *Ddef1*, *Ddx19b (2810457M08Rik)*, *Dedd2*, *Dnmt3a*, *Elk3*, *Hnrpa1*, *Hrb*, *Ifih1*, *Isgf3g*, *Mxd4*, *Nab1*, *Rab8b*, *Rbms2*, *Rnaset2*, *Rpl12*, *Rpo1-1*, *Skil (9130011J04Rik)*, *Sp100*, *Tcf3*, *Tef*, *Trim21*, *Trim30*, *Wasl*, *Zbp1*, *Zcchc7*, *Zfp260*, *Zfp313*, and *Zfp445 (AW610627); *signaling-associated genes *Bin1*, *Dlgh1*, *Myd88*, *Nedd9*, *Pacsin1*, *Pscdbp*, *Sdc3*, *Shkbp1*, *Sytl2*, *Traf1*, *Vps28*, *Xpo6*; and those encoding receptors, such as *1810011E08Rik*, *Brd8*, *Cd9*, *Folr4*, *Ptger2*, *Ptgir*, *Sorl1*, and *Tlr2*. The complete set of genes identified in this category is listed in Additional data file 2.

The next largest pattern categories comprised probesets that were unique to positive selection, consistent with this condition being the most differentiated based on the Euclidean analysis. These categories (Figure 2) included genes that were induced (patterns B and C) or repressed (patterns H and I) during positive selection, but were either not altered at all during negative selection (C and I) or underwent a change of lesser magnitude but in the same direction (B and H). Genes in these categories are candidates for translating TCR engagement by weak agonists into survival and maturation rather than negative selection. However, this category will also include genes that are developmentally regulated at later stages of SP cell maturation, after CD69 induction and increased TCR surface expression, since negative selection will remove SP cells before reaching this stage. Patterns B and C comprised 278 probesets that were preferentially induced during positive selection, including the well established functional genes *CD3ζ *and the calcineurin-response gene *Itgb7*. Patterns H and I comprised 394 probesets that were preferentially repressed during positive selection, including developmental markers *Cxcr4*, *CD8α*, *CD24a*, *CD25*, and cell cycle genes *Cdc2*, *Cdc6*, *Cdc20*, *Cdc25b*, *Cdka2c *and *Myb*.

As well as these previously identified markers of positive selection, a number of important T cell regulatory genes were also found here to be upregulated in positive selection: those encoding nucleic acid binding proteins, such as *Dbp *[33],*Foxo1 *[47] and *Zfp67 *[48]; the signaling-associated gene *Stam2 *[49]; and those encoding receptors, such as *Il6ra *[50] and *Itgb2 *[51].

Previously unassociated genes identified as part of this analysis are, therefore, excellent candidates for novel markers of positive selection, and include those encoding nucleic acid binding proteins, such as *Bhc80*, *Ddb2*, *Ezh1*, *Gata1*, *Pcbp4*, *Rbms1*, *Smarca2*, and *Trim26*, *Ddit3*, *Foxp1*, *Oas2*, *Tgif2*, and *Zfp467*; signaling-associated genes *Arrb1*, *Bcap31*, *Emid1*, *L1cam*, *Numb*, *Pea15*, *Rabip4*, *Rcbtb1*, *Rsn*, *Selpl*, and *Sh3gl1*; and those encoding receptors, such as *AA691260*, *D7Ertd458e*, *Frag1*, *Gpr97 Grina*, *Il6st*, *Paqr7*, *Robo3 *and *Sh2d3c*. The complete list (Additional data file 2) dramatically expands the set of candidate mediators and markers for understanding positive selection and SP cell maturation.

A smaller transcriptome of 246 probesets comprised genes that were preferentially or selectively induced or repressed during negative selection (patterns D, E, F, J, K, L in Figure 2), consistent with negative selection being closer to pre-selection in the global analysis. Genes in these categories are candidate mediators or markers of negative selection and thymocyte apoptosis. Pattern categories E and K were selectively induced or repressed during negative selection, showing no change in expression between pre-selection cells and positive selection. Pattern E comprised 87 probesets, including the TCR-induced pro-apoptotic gene *Bim *(*Bcl2l11*), which has previously been shown to be induced selectively during negative selection in this system and is an essential mediator of negative selection, and activation markers such as *Ccr6*.

Genes in patterns D and J were induced or repressed more strongly in negative than positive selection. Probesets in these categories are likely to include genes that report the quantitative differences in TCR signaling thought to differentiate strong TCR engagement by negative selecting agonists from weak engagement by positively selecting agonists. Pattern D comprised 48 probesets, including the gene encoding the thymocyte apoptosis-inducing transcription factor, *Nur77*, markers of activated T cells or regulatory T cells, *Gitrd*, *Ox40 *and *41bb*, and the ERK-response gene *Fos*. Category F and L comprised a small number of probesets that exhibited a change in expression during negative selection that was in the opposite direction to that induced during positive selection. Pattern L includes genes such as *CD25*, *CD24a *and *Annexin A4*.

Overall, the negative selection transcriptome is quite small: 144 induced probesets, and 102 repressed probesets. By contrast, positive selection of early DP thymocytes into early SP thymocytes induced 809 probesets and repressed 1,086 probesets - a transcriptional program that is eight-fold larger. A complete list of the positive and negative selection transcriptomes, all the genes falling into patterns A to L, in the B10^k ^strain is given in Additional data file 2.

### Genomic localization of gene expression changes induced by selection on the B10^k ^background

Recent studies have found that sets of genes induced by similar stimuli can co-localize in genomic clusters [52]. To determine if positive or negative selection likewise work by the activation or suppression of broad clusters of genes we queried the data to identify cytogenetic bands with a significantly increased proportion of induced or repressed genes, normalized to their gene density.

Focusing first on genes associated with positive selection, there is evidence for a small degree of gene expression change by cytogenetic band. Six cytogenetic bands were broadly suppressed upon positive selection, and no cytogenetic bands were broadly activated. Of the six altered cytogenetic bands, three meet stringent false discovery rates, 12F, 3B and 2F (Table 1).

**Figure 3:**
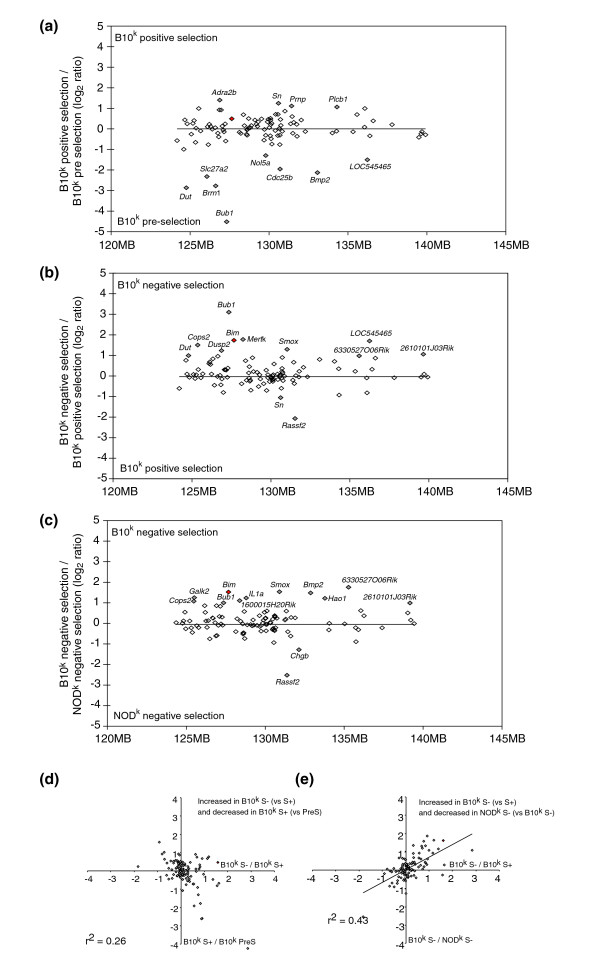
Gene expression changes localized to cytogenetic band 2F. Cytogenetic band 2F was analyzed for the relative expression values of Affymetrix annotated genes. The log_2 _ratio of each gene (diamonds), plotted by genomic position, is displayed for: **(a) **B10^k ^PreS compared to B10^k ^S+; **(b) **B10^k ^S- compared to B10^k ^S+; and **(c) **B10^k ^S- compared to NOD^k ^S-. Comparisons of gene expression changes under these conditions were made by plotting the log_2 _ratios for all genes within cytogenetic band 2F against each other (diamonds) and calculating the r^2 ^value. The distance from the origin thus reflects the degree of expression change. **(d) **Ratio of gene expression in B10^k ^PreS compared to B10^k ^S+ (*y*-axis) versus the ratio of expression in B10^k ^S- compared to B10^k ^S+ (*x*-axis). **(e) **Ratio of gene expression in B10^k ^S- compared to NOD^k ^S- (*y*-axis) versus B10^k ^S- compared to B10^k ^S+ (*x*-axis). In each case the probeset measuring *Bim *expression is indicated with a red diamond. All probesets with a relative change greater than +1 or less than -1 are shaded grey and annotated.

The gene expression changes induced upon negative selection, by contrast, are highly localized. While the global difference (Euclidian distance) between positive selection and negative selection is only half that of positive selection to pre-selection (Figure 1), a greater number of cytogenetic bands show enrichment of gene expression differences. That is, while fewer genes were changed, and with a lesser magnitude, in positive selection when compared to negative selection than in positive selection compared to pre-selection, the genes that were differentially expressed were highly co-localized to certain genomic regions. One region was broadly activated in response to negative selection, 2F, while seven regions were broadly suppressed upon negative selection, of which two meet stringent false discovery rates, 11E and 6B (Table 1). Region 2F is also of interest because it is a region that contains a cluster of genes that decreased in expression during positive selection (Figure 3a), and a cluster of genes that increased in expression during negative selection (Figure 3b). The induced and repressed clusters are, however, largely distinct, with little correlation (Figure 3d). Of significance, the key pro-apoptotic gene *Bim *is encoded within 2F. *Bim *is controlled at least partially by chromatin structure, as histone deacetylase inhibition allows the spontaneous induction of *Bim *followed by apoptosis [53]. Of the 2F probesets changed by negative selection in the B10^k ^strain, the highest level of upregulation is observed in *Dut*, *Cops2*, *Dusp2*, *Bub1*, *Bim*, *Mertk*, *Smox*, *6330527O06Rik*, *LOC545465 *and *2610101J03Rik*. Similarly, the 2F probesets with the greatest defects in upregulation in the NOD^k ^strain are *Cops2*, *Galk2*, *Bub1*, *Bim*, *1600015H20Rik*, *IL1a*, *Smox*, *Bmp2*, *Hao1*, *6330527O06Rik *and *2610101J03Rik*. These data generate the hypothesis that the cytogenetic band 2F contains a concentration of apoptotic initiators for negative selection that may be coregulated by chromatin structure.

### Global gene expression changes induced upon positive and negative thymocyte selection in the NOD^k ^strain

In parallel with the above analyses of negative and positive selection in B10^k ^mice, the same cell subset markers, sorting methods, and mRNA labeling and hybridization to microarrays were applied to pre-selection (PreS), early positive selection (S+) and early negative selection (S-) thymocytes from TCR and TCR insHEL animals on the NOD.*H2*^k ^strain background. This allowed developmentally matched, homogeneous populations of T cells to be traced during positive and negative selection using the same TCR and self peptide-MHC ligands, but carrying all the NOD genomic differences from B10 with the exception of the congenically matched *H2*^k ^haplotype.

The global gene expression differences between pre-selection DP cells and early SP cells undergoing positive or negative selection were first used to compare these states by Euclidian distance (Figure 1). The difference between pre-selection and positive selection thymocytes was similar on the NOD^k ^background (23.4 units) to that observed on the B10^k ^background (26.7). On the NOD background, however, there was much less difference between positive and negative selection, with the Euclidean distance between these states decreased from 13.9 in B10^k ^to 8.9 in NOD.

Individual gene expression differences between pre-selection, positive selection and negative selection on the NOD background were categorized into the same 12 patterns, as conducted above for the equivalent cells from B10 strain animals (Figure 2).

Focusing first on patterns A and G, representing probesets that were induced or repressed equivalently during positive or negative selection, these categories contain the largest number of genes that were induced (787 probesets) or repressed (994 probesets), which are comparable to the numbers observed for these categories in the B10 strain (Figure 2). Again, category A includes genes that are developmentally increased during maturation from DP to SP cells, such as *IL7R*, and genes that are induced by TCR signals during positive and negative selection, such as calcineurin-response genes *Ian1*, *Egr2 *and *CD52 *and ERK-response genes *Nab2 *and *Zfp36l1*. In total, 240 of the probesets assigned to category A in NOD were also assigned to this category in B10. The stringent cut-offs used to assign probesets to pattern categories underestimated the similarity of gene expression during DP to SP maturation on the two strain backgrounds, because only 39 of the 531 pattern A probesets in B10 thymocytes have significantly different values from NOD for the corresponding cell types. Likewise, genes that were decreased during maturation (pattern G) included expected developmentally regulated genes such as *Rag1*, *Cd8α*, *PreTα *and cell cycle genes. Of the 692 B10 pattern G probesets, only 56 have significantly different values to NOD for both positive and negative selection. Thus, the NOD background had little effect on gene expression changes associated with early SP maturation from pre-selection DP cells.

By contrast, the NOD background has markedly reduced numbers of genes with expression patterns that differentiate negative from positive selection (patterns B to F and H to L), consistent with the smaller Euclidean distance between these two states in the global analysis. Thus, of the patterns with increased expression in both positive and negative selection (A, B, D), 79% show the same degree of regulation during positive and negative selection (assigned to pattern A) in B10^k ^mice, but 94% show the same degree of regulation in NOD^k ^mice (with NOD^k ^pattern A containing many probesets assigned to patterns B or C in B10^k ^mice). Likewise, of the patterns with decreased expression in both positive and negative selection (G, H, J), 72% show the same degree of regulation (assigned to pattern G) in B10^k ^mice, but 95% show the same degree of regulation in NOD^k ^mice. By this assessment, positive and negative selection are less distinct in NOD^k ^mice than in B10^k ^mice.

**Figure 4:**
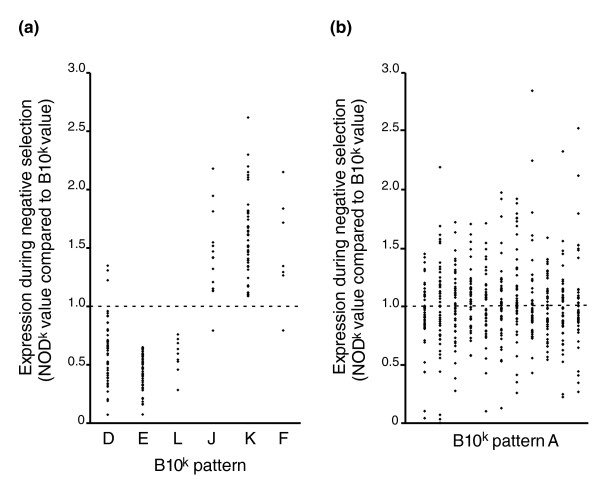
Graphical representation of expression changes between the B10^k ^and NOD^k ^strains. Probesets falling in specific B10^k ^clusters were assessed for the ratio of expression during negative selection in NOD^k^:B10^k ^mice. A value of 1 represents equal expression during negative selection, <1 represents lower expression in NOD^k ^thymocytes than B10^k ^thymocytes during negative selection, and >1 represents higher expression in NOD^k ^thymocytes than B10^k ^thymocytes during negative selection. **(a) **Probeset patterns from the B10^k ^analysis increased (D, E, L) or decreased (J, K, F) specifically during negative selection trended towards showing that the same probeset in NOD^k ^thymocytes had a lower/higher expression, respectively. **(b) **The probeset pattern from the B10^k ^analysis increased during maturation (A) showed roughly the same level of expression during negative selection in NOD^k ^thymocytes (divided into random groups for clarity).

Focusing specifically on genes that were preferentially or selectively induced (patterns D, E, L) or repressed (J, K, F) during negative selection revealed a global dampening of the negative selection response in the NOD background (Figure 4). Patterns D, E, and L, comprising probesets that were induced during negative selection either selectively (E, L) or to higher levels than during positive selection (D), contained only 66 probesets in NOD mice, whereas these sets were more than twice as large (144 probesets) on the B10 background. Moreover, of the 144 B, E or L probesets that were specifically induced during negative selection in B10 mice, 137 were diminished in expression during negative selection in NOD mice, 112 by more than 20% and 82 by a significant amount (Figure 4a). Thus, as noted previously, *Bim *induction (category E in B10) is undetectable in NOD thymocytes, while *Nur77 *induction (category B in B10) is greatly diminished.

Similarly, genes that were selectively decreased in negative selection (patterns J, K, F) accounted for only 46 probesets in the NOD strain compared to 102 in B10. Of the 102 probesets decreased upon negative selection in B10 mice, 100 remained at higher levels during negative selection in NOD^k ^mice, 84 by more than 20% and 44 significantly so (Figure 4a). Combining both transcriptional increases and decreases, of the 246 probesets specifically changed by negative selection in the B10^k ^mouse, 51% were significantly less changed in the NOD^k ^mouse. By contrast, of the 531 pattern A probesets that were increased equivalently during positive and negative selection in the B10^k ^mouse, only 7% had significantly different expression during negative selection in NOD^k ^compared to B10^k ^mice, and the majority showed similar expression (Figure 4b).

**Figure 5:**
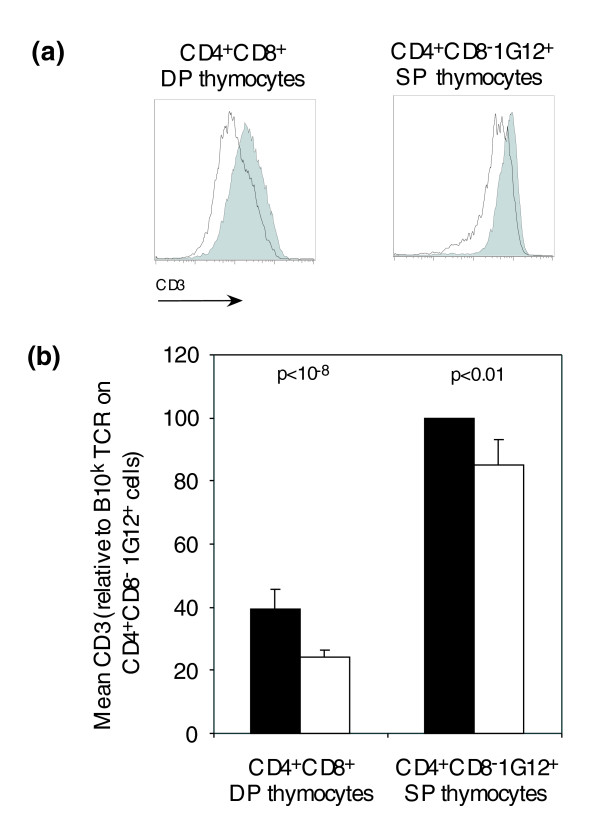
Regulation of surface CD3 levels on 3A9 TCR transgenic thymocytes on the B10^k ^and NOD^k ^genetic backgrounds. 3A9 TCR transgenic mice on the B10^k ^and NOD^k ^backgrounds were assessed for CD3 surface levels by flow cytometry, at both the DP and SP 1G12^+ ^developmental stages. **(a) **Representative histograms for CD3 expression on DP cells and SP 1G12^+ ^cells. B10^k ^mice are shown in grey, NOD^k ^mice in white. **(b) **The mean fluorescence intensity (normalized to B10^k ^SP 1G12^+ ^thymocytes) and standard error is shown with B10^k ^in black and NOD^k ^in white (*n *= 7 for B10 values, 17 for NOD values). Significance of differences between B10^k ^and NOD^k ^groups of the same genotype are indicated by *t*-test *p *values about the group. *t*-tests comparing SP 1G12^+ ^thymocyte expression were tested using a two sided *t*-test of the hypothesis that NOD^k ^expression levels are different to '100'.

The presence of reduced upregulation and downregulation across the entire spectrum of negative selection-specific genes in NOD^k ^thymocytes indicates that upstream effects are at least partially responsible. This observation, recognizable only at a genomic level, was not predicted in previous analyses that focused solely on changes in downstream effectors, such as Bim and Nur77 [10,11]. Such a defect may be occurring at the early signaling synapse, in line with a recognized alteration of TCR signaling components, such as enhanced Fyn kinase activity, differential activation of the Cbl pathway, impairment of membrane-translocation of Son of sevenless (mSOS) Ras GDP releasing factor, and the exclusion of mSOS and Phospholipase C (PLC)-γ1 from the TCR-Grb2-Zap70 complex, resulting in hypoactivation [54]. Altered signaling in the basal TCR apparatus may be responsible for the reduced surface CD3 levels present on TCR transgenic thymocytes (without the presence of insHEL) in the NOD^k ^strain compared to their B10^k ^counterparts, with a 50% reduction at the DP stage, and a 20% reduction at the SP stage (Figure 5).

The NOD background also caused a large decrease in probesets assigned to categories B, C, H, and I, comprising genes that are preferentially or selectively altered during positive selection (Figure 2). This result has two non-exclusive explanations. First, there may be a less efficient positive selection response in NOD. Alternatively, many of the genes in this category may normally be developmentally regulated to appear at later stages of SP cell maturation, before CD69 is lost but at a stage when negative selection would have removed most such cells.

### Constitutive differences in thymocyte gene expression caused by the NOD background

In addition to the altered negative and positive selection response above, the NOD background also had altered pre-selection gene expression in TCR^low^CD69^- ^DP cells, which may set the stage for altered responses when the cells encounter negative selecting antigens. Six independent pools of pre-selection DP cells were analyzed on both B10 and NOD backgrounds: three from TCR animals and three from TCR insHEL animals. There were few differences between TCR and TCR insHEL pre-selection pools within a strain background, consistent with sorting for antigen-nasïve thymocytes that had yet to display TCRs for HEL and induce CD69. Comparing pre-selection cells between the strains at a global level first (Euclidian distance, Figure 1), the difference between these states (14.2) was approximately half that of the difference between pre-selection DP and early positive selection SP cells (26.7), with a total of 1,484 probesets significantly different between NOD PreS and B10 PreS (1,484 probesets). It is unknown if this degree of pre-selection divergence is specific to the NOD^k ^strain, or if it is observed across multiple strains based on comparative divergence.

In terms of genomic location, these changes are particularly concentrated in 20 cytogenetic regions (Table 1). Twelve regions show increased expression in B10^k ^pre-selection thymocytes, eleven of which meet stringent false discovery rates: 8E, 7E, 18E, 12F, 6C, 1B, 15D, 5F, 10C, 19A and 4E. Likewise eight regions show increased activity in NOD^k ^pre-selection thymocytes, all of which meet stringent false discovery rates: XF, 3E, XD, 3H, 5C, 12C, 1A and XA. With regard to the phenomenon of defective negative selection in the NOD^k ^strain, it may be of relevance that two of these regions co-localize with genomic loci that contribute to defective negative selection [10], 7E and 15D.

Gene expression differences between NOD^k ^and B10^k ^strains induced upon negative selection were also analyzed for cytogenetic clustering. Only four cytogenetic bands show enrichment, after eliminating regions changed in the basal (that is, pre-selection thymocytes) state. Two regions, 2F and 3A, were broadly suppressed in the NOD^k ^strain, and two regions were broadly activated in the NOD^k ^strain, neither of which meet stringent false discovery rates (Table 1). The region 2F is of particular interest for several reasons. Firstly, this is the only region that was broadly activated upon negative selection in the B10^k ^strain (Figure 3b). Secondly, it is one of only two regions that show broad strain differences in regulation upon induction of negative selection, with lower activity in the NOD^k ^strain (Figure 3c). Thirdly, this region overlaps one of the six identified loci with a causative effect in defective negative selection in the NOD^k ^strain [10]. A comparative analysis of the gene expression changes in B10^k ^and B10^k^-NOD^k ^negative selection demonstrated that this locus comprises the same cluster of genes that are upregulated upon negative selection in the B10^k ^strain and show poor induction of gene expression in the NOD^k ^strain (Figure 3e). These data indicate that the NOD^k ^strain has a genetic defect preventing the efficient induction of the negative selection 2F cluster, including *Bim*, preventing initiation of apoptosis.

### Gene expression variants representing causal candidates for defective thymic deletion in NOD

Combining the global transcription profiles for NOD^k ^and B10^k ^pre-, positive and negative selection with linkage data for efficiency of negative selection in the same *in vivo *conditions [10] provides a way to identify candidate genes responsible for the NOD trait of defective negative selection. While expression differences are promising candidates for quantitative traits, we recognize that this approach is unable to detect allelic variants arising from differential mRNA splicing, such as the *Idd5 *allele of *Ctla4 *[55], or from amino acid substitutions, such as the *Idd13 *polymorphism in *β2 m *[56].

**Figure 6:**
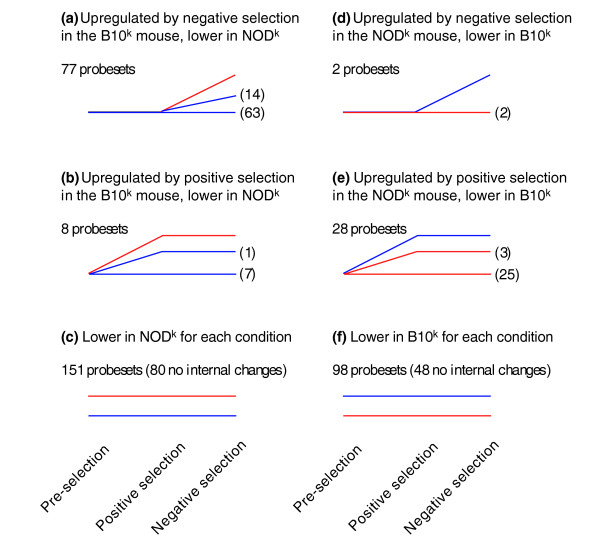
Grouping of key gene changes between NOD^k ^and B10^k ^thymocytes. Analysis of Affymetrix GeneChip data segregated those probesets that were significantly modified in a 2 × 2 × 2 F-test (*p *< 0.005) into groups 1 to 6 based on logical sets of significant contrasts (*p *< 0.05). The diagrams show relative changes for the mean gene expression in the pre-selection (PreS), positive selection (S+) and negative selection (S-) conditions. The number of probesets falling into each group is listed on the group pattern. B10^k ^expression changes are shown in red, NOD^k ^expression changes are shown in blue.

Expression pattern was first used to identify six categories of high priority candidate genes (Figure 6, Additional data file 2). Group 1 consists of probesets that were preferentially increased in B10^k ^negatively selecting thymocytes (patterns D, E and L), but were significantly less increased in NOD^k ^counterparts. There are 77 probesets in this group, of which 14 show poor induction and 63 show no induction. Defective apoptotic initiators could fall in this group. Group 2 consists of probesets that were increased in B10^k ^thymocytes upon selection (pattern A), but were significantly less increased in NOD^k ^mice (p < 0.05). Only 8 probesets are in this group, of which seven show no increase upon maturation in the NOD^k ^strain. Defective functional prerequisites for negative selection switched on during positive selection could fall in this category. Group 3 consists of probesets that were significantly lower in NOD^k ^thymocytes compared to B10^k ^thymocytes for each biological condition. There are 151 probesets in this group, 80 of which show no development or selection-induced differences within the NOD^k ^or B10^k ^groups. Defective constitutively expressed prerequisites for negative selection could fall in this group. Group 4 is the reverse of group 1. It consists of probesets that were increased in the NOD^k ^negatively selecting thymocytes, but were significantly less increased in B10^k ^thymocytes. This group is designed to catch candidate genes that were more strongly induced in NOD^k ^negatively selecting thymocytes and provided protection from negative selection. Only two probesets fall in this category. Group 5 is the reverse of group 2. It consists of probesets that were increased in NOD^k ^thymocytes upon selection (pattern A), but were significantly less increased in B10^k ^thymocytes. This group is designed to catch NOD^k ^over-expressed maturation-induced protective genes. Group 6 is the reverse of group 3, comprising probesets that were significantly higher in NOD^k ^thymocytes compared to B10^k ^thymocytes for each biological condition. There are 98 probesets in this group, 48 of which show no developmental or selection-induced differences within NOD^k ^or B10^k ^thymocytes. Constitutively over-expressed protective factors could fall in this group.

A matrix comprising the probesets in these six categories and the genomic location to a 30 cM bracket surrounding peak linkage to the four regions of NOD^k ^susceptibility to defective negative selection markers (*D7mit101*, *D15mit229*, *D2mit490/Idd13 *and *D1mit181/Idd5*) [10] identified 44 candidate probesets. Each region has 6 to 10 candidates using this method, except for the region centered on *D2mit490*, which includes the 2F cluster and has 20 candidates. These are discussed in more detail below, as summarized in Table 2.

**Figure 7:**
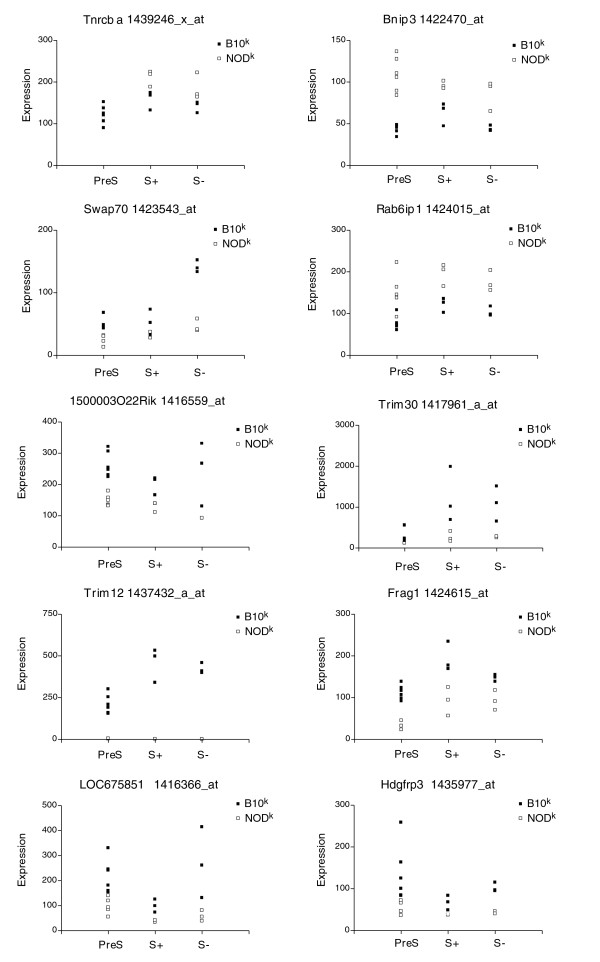
Expression of candidate genes for the *D7mit101*-linked defective thymic deletion allele. Individual values of biological replicates from the Affymetrix 430A microarray chips are indicated in the biological groups of 'pre-selection' (PreS), 'positive selection' (S+) and 'negative selection' (S-). Values are represented in black (B10^k^) and white (NOD^k^) boxes.

Of the candidate genes linked to the *D7mit101 *loci (Ch7, 60 cM), four genes are of particular interest (Figure 7, Table 3). *Bnip3*, 8.4 cM from *D7mit101*, was approximately two-fold over-expressed in NOD^k ^thymocytes in every condition. Bnip3 is a BH3-only protein that dimerizes with Bcl-X(L), making a pro-apoptotic heterodimer [57,58]. Bnip3 has been shown to translocate to the mitochondria to induce apoptosis during CD47-induced apoptosis [59], nitric oxide induced apoptosis in macrophages [60], hypoxic apoptosis [61], and activation induced death of cytotoxic T cells [62]. Overexpression of *Bnip3 *would, therefore, be unlikely to protect NOD^k ^thymocytes from clonal deletion. *Bnip3 *overexpression may instead be a downstream effect of the NOD defective thymic deletion allowing thymocytes to tolerate a higher level of expression, just as *Bnip3 *overexpression is associated with more aggressive tumors and poor survival in human patients [63]. *Trim30 *and *Trim12 *are approximately 13 cM from *D7mit101 *and were poorly expressed in NOD^k ^thymocytes. Trim30 shows an average of a 2- to 3-fold decrease in expression in NOD^k ^thymocytes (represented by three probesets, two of which show a strong difference and a third in the 5' untranslated region (UTR) that shows little difference), while Trim12 shows an approximately 300-fold expression decrease in NOD^k ^thymocytes (Figure 7). Both are members of the tripartite motif family, with RING, B-box type 1 and 2, and coiled coil domains [64]. Little is known about their function, but the extent of the change and putative domain function make them strong candidate genes.

**Figure 8:**
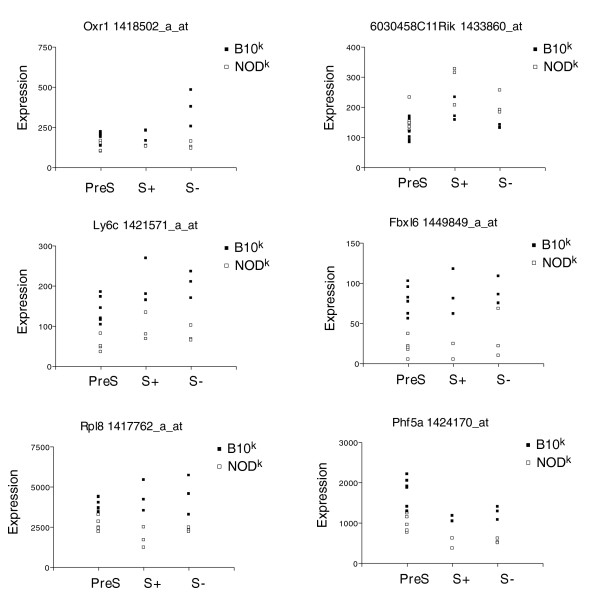
Expression of candidate genes for *D15mit229*-linked defective thymic deletion. Individual values of biological replicates from the Affymetrix 430A microarray chips are indicated in the biological groups of 'pre-selection' (PreS), 'positive selection' (S+) and 'negative selection' (S-). Values are represented in black (B10^k^) and white (NOD^k^) boxes.

A key candidate gene for the *D15mit229*-linked defective thymic deletion loci (Ch15, 22 cM; Figure 8, Table 4) is *Ly6c*. *Ly6c *is 21.1 cM from *D15mit229*, and was poorly expressed in NOD thymocytes under all conditions (2.3-fold decrease; Figure 8). This reduced expression has been previously observed to be due to a *Ly6c *promoter polymorphism in NOD mice, and is thus a known *cis *effect [65]. Functionally, Ly6c inhibits the signal for secretion of interleukin (IL)2 and proliferation in peripheral CD4^+ ^cells [65], and cross-linking of Ly6c causes clustering of Leukocyte function associated molecule 1 (LFA-1, CD11a/CD18) on the surface of CD8 T cells [66].

**Figure 9:**
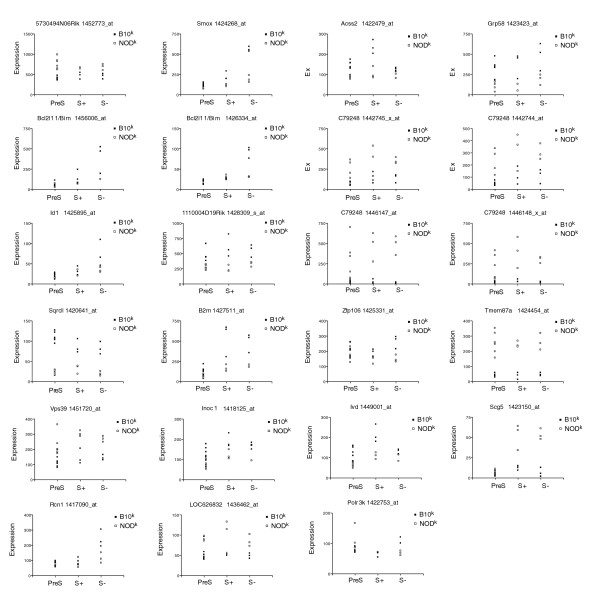
Expression of candidate genes for *D2mit490*-linked defective thymic deletion. Individual values of biological replicates from the Affymetrix 430A microarray chips are indicated in the biological groups of 'pre-selection' (PreS), 'positive selection' (S+) and 'negative selection' (S-). Values are represented in black (B10^k^) and white (NOD^k^) boxes.

Many candidate genes were identified in the 2F cytogenetic band around *D2mit490*- (Ch2, 65 cM), of which four are of particular interest (Figure 9, Table 5). Smox oxidizes spermine to spermidine [67], which has been shown to induce DNA damage and apoptosis in gastric epithelial cells [68]. The major control for Smox during polyamine-induced apoptosis has been shown to occur at the mRNA level [69]; therefore, the small increase during B10^k ^positive selection and large increase in negative selection could prepare B10^k ^thymocytes for apoptosis (Figure 9). The lower levels of *Smox *in NOD^k ^thymocytes, and the negligible increase during negative selection, could contribute to a poor apoptotic response. *Bim/Bcl2l11 *as a candidate gene has been previously validated [10], having a key role in negative selection [70,71] and defective upregulation in the NOD^k ^strain (in three probesets; two probesets in the 3' UTR region of one splice variant showed little change). Another candidate gene in this region is *Id1*, 8.8 cM from *D2mit490*. It was increased during negative selection 2.2-fold higher in B10^k ^thymocytes than NOD^k ^thymocytes (Figure 9), and has been shown to inhibit the function of E2A and Transcription factor 12 (HEB), increasing the response to TCR stimulation and the sensitivity of thymocytes to apoptosis [72]. Thus, *Id1 *induction may be required to amplify the clonal deletion signal for apoptosis. *Pdia3*, 10.9 cM from *D2mit490*, was expressed at 3.4-fold higher levels in B10^k ^thymocytes (Figure 9). Its function as an endoplasmic reticulum chaperone required for mitomycin C-induced death [73] indicates potential candidacy. Also of interest is *Pdrg1*, 8.8 cM from *D2mit490*, which has previously been shown to be upregulated in response to UV radiation and inhibited by p53 [74]. It is of interest because of the basal low level observed in NOD^k ^thymocytes.

**Figure 10:**
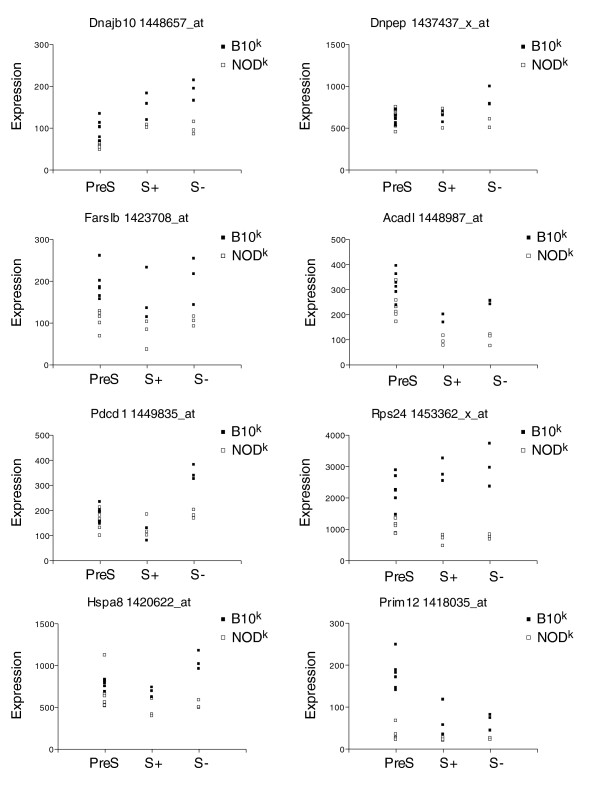
Expression of candidates for the defective thymic deletion allele linked to *D1mit181*. Individual values of biological replicates from the Affymetrix 430A microarray chips are indicated in the biological groups of 'pre-selection' (PreS), 'positive selection' (S+) and 'negative selection' (S-). Values are represented in black (B10^k^) and white (NOD^k^) boxes.

The fourth NOD locus contributing to defective negative selection in TCR insHEL mice, linked to *D1mit181 *(Ch1, 43 cM), includes the genes *Ctla4 *and *Pdcd1 *(Figure 10, Table 6). The *Ctla4 *gene was not differentially expressed in our analysis, but it is a functional candidate because *Ctla4*-deficient thymocytes are resistant to radiation-induced apoptosis [75]. The NOD *Idd5 *(*D1mit181*-linked) haplotype produces less of an alternatively spliced *Ctla4 *gene product that is a constitutively active inhibitor of TCR zeta phosphorylation [55,76]. The orthologous region in humans also contains a *CTLA4 *variant and has been associated with diabetes, thyroid autoimmunity, Addison's disease, and disease severity in multiple sclerosis [55]. As mentioned during the preliminary analysis [10], *Pdcd1 *was induced during negative selection in B10 but not in NOD thymocytes (Figure 10). Pdcd1 (also called PD1) has previously identified functions in negative regulation of T cell function [77] and *Pdcd1*^*0/0 *^mice display reduced positive selection [78] and autoimmune symptoms [79,80]. Furthermore, blockade of Pdcd1 induces diabetes in NOD mice [81].

The analysis of the negative selection transcriptome here distinguishes among several distinct, but not mutually exclusive, mechanisms accounting for defective negative selection in NOD thymocytes: first, several downstream effectors, such as Bim, are defective; second, the entire negative selection induction process is reduced; third, there is a broad defect in the induction of TCR signaling response genes (both positive and negative selection); and fourth, the NOD^k ^strain induces an additional, protective transcriptome during negative selection. By comparison of the NOD^k ^strain to the B10^k ^strain at a global transcription level, there is no evidence for the ectopic of an additional, 'protective' gene set, nor for an obvious defect in the TCR signaling-dependent process of positive selection. The second possible mechanism appears correct, as there was a global reduction by approximately 40% in the transcriptional process of negative selection, indicating that a defect in upstream events impacted on multiple downstream mediators. Not exclusive from the upstream defect, several important apoptosis effector genes, including *Bim*, are almost completely absent from the NOD negative selection response, raising the possibility that these genes are at points where individual quantitative differences summate, with *cis*-acting promoter defects having an additive effect with the defect in upstream inductive events. In particular, the cluster of poorly induced negative selection genes in cytogenetic band 2F around *Bim *raises the possibility of a *cis*-acting allelic variation contributing to poor induction of this locus. Of interest, the defect is not absolute, with partial upregulation seen for the majority of the negative selection gene set, correlating with previous cellular data indicating that NOD thymocytes are capable of strong negative selection when exposed to higher levels of stimuli [10].

The candidate genes discussed above act at multiple points in the pathway between binding to TCR of negatively selecting peptide-MHC and triggering of thymocyte apoptosis. These data frame a hypothesis that defective negative selection involves the summation of many incremental decreases in the efficiency of this signaling pathway, caused by multiple allelic variants at four chromosomal loci. In NOD thymocytes there is lower surface TCR expression, and lower efficiency of TCR signaling due to increased *iCTLA4 *and decreased *Pdcd1 *and *Id1*. This general decrease in TCR signaling may then be compounded by poor inducibility or low expression of apoptosis inducers *Bim*, *Smox*, *Bnip3*, and *Pdia3*. By producing a molecular map of negative selection responses *in vivo*, the results from this study open up pathways to understand the mechanism of negative selection and the basis for its quantitative variation leading to autoimmune disease.

## Materials and methods

The data discussed in this publication have been deposited in the NCBI Gene Expression Omnibus [20], as MIAME compliant data, and are accessible through GEO Series accession number GSE3997.

### Microarray preparation

As previously described [10], cell populations were purified from healthy 6-8-week old female mice, stained in 5 μg/ml actinomycin D and 2 μg/ml α-amanitin (Sigma-Aldrich, St Louis, MO) with CD4- fluorescein isothiocyanate (FITC), CD69-phosphatidylethanolamine (PE), CD8- peridinin chlorophyll protein (PerCP) and 1G12 indirectly labeled with Allophycocyanin (APC), then sorted with a Becton Dickinson (Franklin Lakes, NJ) FACVantage. Sorted populations were 'early DP' (CD4^+^CD8^+^CD69^-^1G12^-^) and 'early SP' (CD4^+^CD8^low^CD69^+^1G12^+^). Purified RNA underwent two rounds of *in vitro *RNA amplification before fragmentation and hybridization to Affymetrix GeneChip 430A arrays (Santa Clara, CA, USA).

### Microarray data processing

The Affymetrix 430A chips were scanned using standard Affymetrix protocols. All arrays passed routine quality control assessment for hybridization and data quality. Expression values, referred to as probeset 'signal', were calculated using the Affymetrix GeneChip analysis software MAS 5.0, with a scaling chosen so that each array has a trimmed mean of 150. Following examination, the endogenous control probesets were removed. The MAS 5.0 signal values were then transformed to logarithms (base 2). Signal values of 0 were assigned a value of 0.1 before taking logs. Finally, the arrays were standardized to mean zero and variance one. Initial gene filtering kept only those probesets that were called 'present' by the Affymetrix signal detection algorithm in all replicates for at least one biological group. All statistical analyses were carried out using these transformed signal values. Statistical analysis was carried out in three data sets, one consisting only of the B10^k ^biological groups, one consisting only of the NOD^k ^biological groups, and one consisting of both B10^k ^and NOD^k ^biological groups.

### Assignment of probesets into gene expression patterns

Assignment of probesets to different patterns of gene expression was separately carried out on the B10^k ^and NOD^k ^datasets. The patterns are defined in terms of direction of change between means, rather than the extent of change, with assignment of a change having a statistical cut-off rather than a fold-change cut-off. For the separate B10^k ^and NOD^k ^datasets, two way analyses of variance (ANOVAs) were performed on each of the selected probesets. For each probeset, if the overall F-test for a test of mean difference was significant (*p *< 0.005), the probeset was considered significantly changed. Having established that the means were different, *t*-tests for the contrasts in means were used to determine significantly different means. A significance level of less than 0.05 was used for these tests. Contrasts between 'pre-selection' thymocytes (CD4^+^CD8^+^1G12^- ^CD69^-^) from TCR transgenic and TCR insHEL double transgenic mice showed very few differentially expressed probesets, as is expected for populations that have not been exposed to HEL antigen due to low expression of TCR and anatomical segregation of antigen in the corticomedullary junction. As a consequence, these two groups were merged and the model re-estimated for all the selected probesets. Since there are now three groups, there are twelve distinct patterns of up, down and no difference. Contrasts between the means using the re-estimated models were used to assign probesets to a gene expression pattern, based on a logical set of significant changes between the various conditions. A three-way ANOVA model was used to analyze the combined B10^k^-NOD^k ^dataset for differential expression between strains. The *p *values are used in this research as indicative of 'evidence' and, except in rare instances, will not be an exact measure of probability. Model assumptions for the use of the different tests were examined for a small number of significant probesets and found to hold. Following analysis, each probeset was annotated for genomic location and gene function using the FACTS (Functional Association/Annotation of cDNA Clones from Text/Sequence Sources) program [82].

### Global gene expression differences

Euclidian distance is the most common measure of metric distance, which is an approximation of the 'distance' between gene expression from replicates in one condition to replicates in other conditions. Euclidean distance is calculated by treating the expression of a group of probesets as a point in n-dimensional space, where the distance from the axis in each dimension represents the expression (the technical mean of transformed signal value for the replicates) of a single probeset. This produces a single point in n dimensions (where n is the number of probesets in the group) that represents one set of conditions, and a single point in the same n-dimensional space that represents the same group of probesets under different conditions. The Euclidean distance between each point (representing each condition) was calculated by using the square root of the sums of squared differences between the points in each dimension, using the formula:

Euclidean distance = √(x_1 _- y_1_)^2 ^+ (x_2 _- y_2_)^2^+ (x_3 _- y_3_)^2 ^+ ...

where x is the point representing the probeset group in condition x, with each dimension x_1_, x_2_, x_3_, and so on being the expression of individual probesets within the group, and y is the point representing the probeset group in condition y, with each dimension y_1_, y_2_, y_3_, and so on being the expression of individual probesets within the group [21].

The straight line distance between the two points thus calculated represents the difference in expression of the included probesets between the two conditions. The probeset group used to calculate the Euclidean distance here included all probesets present in all replicates for at least one condition, thus creating an approximation of the genome-wide similarity between two conditions [21,83,84].

### Genomic clustering analysis

Gene Set Enrichment Analysis (GSEA) was used to examine the genome for areas of differential expression by cytogenetic band. GSEA R script (script defaults, standard method) [85] was used with the 'gene label' permutation method. Genes represented by multiple probesets were reduced to a single probeset with the smallest overall *p *value. A lower cut-off of 20 genes per cytogenetic band was used, which resulted in a total of 113 gene sets being tested for enrichment. A false discovery rate cut-off of 0.4 was initially used, with the stricter criterion of 0.25 for regions listed. Regions with enriched gene expression during positive selection (B10^k^) refer to cytogenetic bands with an enriched number of genes with expression differences between B10^k ^pre-selection thymocytes and B10^k ^positive selection thymocytes (TCR transgenic early SP cells). Regions with enriched gene expression during negative selection (B10^k^) refer to cytogenetic bands with an enriched number of genes with expression differences between B10^k ^positive selection thymocytes (TCR transgenic early SP cells) and B10^k ^negative selection thymocytes (insHEL:TCR double transgenic early SP cells). Regions with enriched strain differences in pre-selection thymocytes refer to cytogenetic bands with an enriched number of genes with expression differences between B10^k ^pre-selection thymocytes and NOD^k ^pre-selection thymocytes. Regions with enriched strain differences induced during negative selection refer to cytogenetic bands with an enriched number of genes with expression differences between B10^k ^negative selection thymocytes (insHEL:TCR double transgenic early SP cells) and NOD^k ^negative selection thymocytes (insHEL:TCR double transgenic early SP cells), with any regions showing enrichment at the pre-selection thymocyte stage removed.

### Flow cytometry

We analyzed 6-10-week old mice (or 6 week post-reconstitution chimeric mice) as described previously [5,10] using the following antibodies: 1G12 anti-clonotype [86] (gift of E Unanue and D Peterson, Washington University, St Louis, MO, USA) culture supernatant followed by rat anti-mouse IgG1 allo-phycocyanin; anti-CD8α-PerCP; anti-CD4-FITC or PE; anti-Ly5a-FITC; anti-CD3-PE; and anti-B220-PE (all from BD PharMingen, San Jose, CA, USA).

## Additional data files

The following additional data are available with the online version of this paper. Additional data file 1 provides a complete listing of statistically significant gene expression changes. In the 'Statistics and annotation' worksheet, basic annotation data are given for each differentially expressed probeset, with the Affymetrix ID number, the gene symbol, chromosome number and starting/ending location, cytogenetic band, gene ontology/molecular function and the Affymetrix target description. Means and gene expression values are given on the transformed scale. The worksheet also contains information on the significance level of analysis of variance tests. The tests were conducted on the transformed gene expression values for each probeset separately. The *p *value for significant differences between conditions is listed if the parent *p *value is less than 0.05. Only genes that showed a significant difference between all the means on this scale, using a threshold of *p *≤ 0.005, are included in the worksheet. 'Within B10' tests for differences within the B10^k ^conditions only (using only B10^k ^condition variance data), with subtests 'Within pre' comparing the B10^k ^pre-selection population sorted from insHEL transgenic and non-HEL transgenic hosts, 'PreS vs S-' comparing B10^k ^pre-selection populations to B10^k ^negative selection populations, 'PreS vs S+' comparing B10^k ^pre-selection populations to B10^k ^positive selection populations, and 'S+ vs S-' comparing B10^k ^positive selection populations to B10^k ^negative selection populations. 'Within NOD' tests for differences within the NOD^k ^conditions only (using only NOD^k ^condition variance data), with subtests performed as per the B10^k ^tests. 'Overall sig' tests for differences between any conditions, with subtests comparing B10^k ^versus NOD^k ^populations at the pre-selection stage ('PreS'), during negative selection ('S-') and during positive selection ('S+'). For each probeset the average expression is given as log_2 _scaled and normalized and arithmetic normalized data, for each condition. Individual data for arithmetic normalized expression are also given for each replicate (PreS 1, 2 and 3 come from non-HEL transgenic hosts, 4, 5 and 6 come from insHEL transgenic hosts). Individual Affymetrix 'Present/Moderate/Absent' calls are given for each replicate. In the 'Raw data' worksheet the individual data for arithmetic normalized expression are given for each probeset, regardless of statistical analysis, along with the Affymetrix target description. Additional data 2 lists the assignment of probesets to expression patterns. The 'B10' worksheet contains information on the expression profiles (patterns) for all probesets that showed a significant difference between the means on the transformed scale, using a threshold of *p *< 0.05 for the B10^k ^strain. Along with the Affymetrix ID are listed the gene symbol, the pattern to which the probeset is assigned in the B10^k ^strain, the pattern to which the probeset is assigned in the NOD^k ^strain, the average arithmetic (unlogged) Affymetrix MAS 5.0 signal values for each condition in the B10^k ^and NOD^k ^strains of pre-selection ('PreS'), positive selection ('S+') and negative selection ('S-'), and the annotated molecular function. In the 'NOD' worksheet, all probesets that meet the significant cut-off for assignment to an expression pattern in the NOD^k ^strain are listed, in the same manner as the 'B10' worksheet. In the 'B10 vs NOD' worksheet, all probesets that meet the significant cut-off for assignment to a differential expression group between the B10^k ^and NOD^k ^strains are listed. Along with Affymetrix ID and gene symbol are listed the group number, the fold-change between the relevant B10^k ^and NOD^k ^conditions (dependent on differential expression group), the *p *values for differential expression between B10^k ^and NOD^k ^strains for each condition, the average arithmetic normalized expression for each condition in the B10^k ^and NOD^k ^strains, and the annotated molecular function.

## Supplementary Material

Additional data file 1 In the 'Statistics and annotation' worksheet, basic annotation data are given for each differentially expressed probeset, with the Affymetrix ID number, the gene symbol, chromosome number and starting/ending location, cytogenetic band, gene ontology/molecular function and the Affymetrix target description. Means and gene expression values are given on the transformed scale. The worksheet also contains information on the significance level of analysis of variance tests. The tests were conducted on the transformed gene expression values for each probeset separately. The *p *value for significant differences between conditions is listed if the parent *p *value is less than 0.05. Only genes that showed a significant difference between all the means on this scale, using a threshold of *p *≤ 0.005, are included in the worksheet. 'Within B10' tests for differences within the B10^k ^conditions only (using only B10^k ^condition variance data), with subtests 'Within pre' comparing the B10^k ^pre-selection population sorted from insHEL transgenic and non-HEL transgenic hosts, 'PreS vs S-' comparing B10^k ^pre-selection populations to B10^k ^negative selection populations, 'PreS vs S+' comparing B10^k ^pre-selection populations to B10^k ^positive selection populations, and 'S+ vs S-' comparing B10^k ^positive selection populations to B10^k ^negative selection populations. 'Within NOD' tests for differences within the NOD^k ^conditions only (using only NOD^k ^condition variance data), with subtests performed as per the B10^k ^tests. 'Overall sig' tests for differences between any conditions, with subtests comparing B10^k ^versus NOD^k ^populations at the pre-selection stage ('PreS'), during negative selection ('S-') and during positive selection ('S+'). For each probeset the average expression is given as log_2 _scaled and normalized and arithmetic normalized data, for each condition. Individual data for arithmetic normalized expression are also given for each replicate (PreS 1, 2 and 3 come from non-HEL transgenic hosts, 4, 5 and 6 come from insHEL transgenic hosts). Individual Affymetrix 'Present/Moderate/Absent' calls are given for each replicate. In the 'Raw data' worksheet the individual data for arithmetic normalized expression are given for each probeset, regardless of statistical analysis, along with the Affymetrix target description Click here for file

Additional data file 2 The 'B10' worksheet contains information on the expression profiles (patterns) for all probesets that showed a significant difference between the means on the transformed scale, using a threshold of *p *< 0.05 for the B10^k ^strain. Along with the Affymetrix ID are listed the gene symbol, the pattern to which the probeset is assigned in the B10^k ^strain, the pattern to which the probeset is assigned in the NOD^k ^strain, the average arithmetic (unlogged) Affymetrix MAS 5.0 signal values for each condition in the B10^k ^and NOD^k ^strains of pre-selection ('PreS'), positive selection ('S+') and negative selection ('S-'), and the annotated molecular function. In the 'NOD' worksheet, all probesets that meet the significant cut-off for assignment to an expression pattern in the NOD^k ^strain are listed, in the same manner as the 'B10' worksheet. In the 'B10 vs NOD' worksheet, all probesets that meet the significant cut-off for assignment to a differential expression group between the B10^k ^and NOD^k ^strains are listed. Along with Affymetrix ID and gene symbol are listed the group number, the fold-change between the relevant B10^k ^and NOD^k ^conditions (dependent on differential expression group), the *p *values for differential expression between B10^k ^and NOD^k ^strains for each condition, the average arithmetic normalized expression for each condition in the B10^k ^and NOD^k ^strains, and the annotated molecular function Click here for file

## Figures and Tables

**Table 1:** Genomic regions with enriched gene expression changes

Cytogenetic band	Size*	NES^†^	NOM *p *val^‡^	FDR q val^§^
Regions enriched for gene expression changes during positive selection (B10^k^)				
Decreased in positive selection				
chr12 F	69	1.8	0.002	0.05
chr3 B	29	1.5	0.035	0.24
chr2 F	100	1.5	0.009	0.24
Regions enriched for gene expression changes during negative selection (B10^k^)				
Increased in negative selection				
chr2 F	100	1.7	0.002	0.08
Decreased in negative selection				
chr11 E	206	-1.7	0.000	0.06
chr6 B	138	-1.5	0.000	0.24
Regions enriched for expression differences between strains in pre-selection thymocytes				
Increased in B10^k^				
chr8 E	110	1.6	0.003	0.17
chr7 E	103	1.6	0.000	0.13
chr18 E	91	1.6	0.008	0.13
chr12 F	69	1.5	0.012	0.14
chr6 C	101	1.5	0.010	0.12
chr1 B	48	1.5	0.030	0.16
chr15 D	99	1.5	0.011	0.14
chr5 F	175	1.4	0.018	0.22
chr10 C	204	1.4	0.007	0.22
chr19 A	199	1.4	0.009	0.21
chr4 E	126	1.4	0.030	0.25
Decreased in B10^k^				
chrX F	109	-2.4	0.000	0.00
chr3 E	43	-1.9	0.003	0.01
chrX D	25	-1.8	0.004	0.01
chr3 H	76	-1.8	0.003	0.01
chr5 C	66	-1.7	0.000	0.03
chr12 C	101	-1.5	0.008	0.09
chr1 A	39	-1.5	0.017	0.09
chrX A	207	-1.5	0.010	0.13
Regions with enriched strain expression differences during negative selection				
Increased in B10^k^				
chr2 F	100	1.8	0.002	0.05
chr3 A	61	1.6	0.008	0.19

*Size: number of genes in the cytogenetic band represented by Affymetrix probesets. ^†^NES: normalized (multiplicative rescaling) enrichment score. ^‡^NOM *p *val: nominal *p *value from the null distribution of the gene set. ^§^FDR q val: false discovery rate q values (only false discovery rate < 0.25 have been included).

**Table 2:** Summary of candidate genes by loci and expression change group

	*D7mit101*	*D15mit229*	*D2mit490*	*D1mit181*
Group	Ch7, 60 cM	Ch15, 22 cM	Ch2, 65 cM	Ch1, 43 cM
1	1	1	5	3
2				
3	6	4	9	5
4				
5	1		1	
6	2	1	5	

**Table 3:** High quality candidates for the *D7mit101*-linked allele

Gene	Probeset	Group	Distance (cM)	Fold change	Function
*Tnrcba*	1439246_x_a	5	6.2	1.3	Unknown
*Bnip3*	1422470_at	6	8.4	2.0	Vital for CD47-induced apoptosis [59], activation induced apoptosis [62], hypoxic apoptosis [61]
*Swap70*	1423543_at	1	9.8	3.1	Part of Ig recombination complex [87], Rac-GEF required for membrane ruffling [88]
*Rab6ip1*	1424015_at	6	10.0	1.8	Interacts with Rab6 [89] (Golgi-ER transport)
1500003O22Rik	1416559_at	3	12.4	1.9	unknown
*Trim30*	1417961_a_at	3	13.2	3.6	RING, B-box type 1 and 2, coiled coil domains [64]
*Trim12*	1437432_a_at	3	13.3	297	RING, B-box type 1 and 2, coiled coil domains [64]
*Frag1*	1424615_at	3	14.6	2.5	Potently activates fibroblast growth factor receptor [90]
LOC675851	1416366_at	3	17.8	3.1	Unknown
*Hdgfrp3*	1435977_at	3	19.6	2.1	Unknown

ER, endoplasmic reticulum. GEF, guanine nucleotide exchange factor.

**Table 4:** High quality candidates for *D15mit229*-linked defective thymic deletion

Gene	Probeset	Group	Distance (cM)	Fold change	Function
*Oxr1*	1418502_a_at	1	0.1	2.7	Protection against oxidative damage [91]
6030458C11Rik	1433860_at	6	18.1	1.5	Unknown
*Ly6c*	1421571_a_at	3	21.1	2.3	Inhibits signal for secretion of IL2 and proliferation [65], cross-linking causes clustering of LFA-1 [66]
*Fbxl6*	1449849_a_at	3	22.0	3.1	Unknown
*Rpl8*	1417762_a_at	3	22.3	1.9	Structural constituent of ribosomes
*Phf5a*	1424170_at	3	25.4	2.1	Nuclear protein, possibly chromatin binding [92]

LFA, Leukocyte function associated molecule.

**Table 5:** High quality candidates for *D2mit490*-linked defective thymic deletion

Gene	Probeset	Group	Distance (cM)	Fold change	Function
5730494N06Rik	1452773_at	6	4.2	1.6	Unknown
*Smox*	1424268_at	1, 3	4.6	2.9	Oxidizes spermine to spermidine [67], resulting in DNA damage and apoptosis [68]
*Bcl2l11/Bim*	1456006_at 1426334_a_at	1	6.7	2.3 3.0	Pro-apoptotic initiator for cytokine deprivation [70] and TCR-stimulation of thymocytes [71]
*Id1*	1425895_a_at	1	8.8	2.2	Inhibits function of E2A and HEB, increases response to TCR stimulation, increases sensitivity to thymocyte apoptosis [72]
*Pdrg1*	1428309_s_at	3	8.8	1.9	Upregulated by UV radiation and downregulated by p53 [74]
*Sqrdl*	1420641_a_at	3	10.1	3.9	Disulfide oxidoreductase activity
*B2 m*	1427511_at	3	10.5	2.7	Component of MHC class I
*Acss2*	1422479_at	3	10.4	1.7	Oxidation of acetate for acetyl-CoA production (utilized mainly for oxidation) [93]
*Pdia3*	1423423_at	3	11.0	3.4	Chaperone in the ER lumen, required for mitomycin C-induced cell death [73]
C79248	1442744_at	6	11.0	2.6	Unknown
	1442745_x_at			3.1	
	1446148_x_at			20.5	
	1446147_at			11.3	
*Zfp106*	1425331_at	3	11.5	1.5	Interacts with FYN and Grb-2 [94]
*Tmem87a*	1424454_at	6	11.6	6.0	Unknown
*Vps39*	1451720_at	6	11.6	2.0	Promotes lysosome clustering and fusion *in vivo*, downstream of rab7 [95]
*Inoc1*	1418125_at	3	12.2	1.6	Unknown
*Ivd*	1449001_at	3	12.5	1.8	Isovaleryl coenzyme A dehydrogenase
*Scg5*	1423150_at	5	15.6	5.6	Neuroendocrine secretory protein [96]
*Rcn1*	1417090_at	1	20.9	2.1	ER Ca^++ ^binding protein [97]
LOC626832	1436462_at	6	22.8	1.9	Unknown
*Polr3k*	1422753_a_at	1	26.8	1.6	RNA polymerase

ER, endoplasmic reticulum. Fyn, fyn proto-oncogene. Grb, growth factor receptor binding protein. HEB, transcription factor 12.

**Table 6:** High quality candidates for the defective thymic deletion allele linked to *D1mit181*

Gene	Probeset	Group	Distance (cM)	Fold change	Function
*Dnajb10*	1448657_a_at	3	0.5	1.7	Homology to heat shock protein DnaJ (Hsp40)
*Dnpep*	1437437_x_at	1	0.5	1.6	Putative aspartyl aminopeptidase function
*Farslb*	1423708_a_at	3	2.5	2.0	Phenylalanine-tRNA synthetase-like, beta subunit
*Acadl*	1448987_at	3	4.8	1.9	Involved in the initial step of mitochondrial beta-oxidation of straight-chain fatty acids
*Pdcd1*	1449835_at	1	11.8	1.9	Negative regulator of T cell function [77], reduces positive selection [78], blockade induces diabetes in NOD mice [81], KO has autoimmune symptoms [79,80]
*Rps24*	1453362_x_at	3	12.0	3.5	Ribosome constituent
*Hspa8*	1420622_a_at	1	17.6	2.0	Aid in the targeting of the MHC class II complex to endocytic compartments [98], regulates cell cycle [99]
*Prim2*	1418035_a_at	3	25.7	3.7	DNA primase [100] involved in primer initiation, elongation, and counting [101]

KO, knockout.
